# Systematic Review of Human and Animal Studies Examining the Efficacy and Safety of *N-*Acetylcysteine (NAC) and *N-*Acetylcysteine Amide (NACA) in Traumatic Brain Injury: Impact on Neurofunctional Outcome and Biomarkers of Oxidative Stress and Inflammation

**DOI:** 10.3389/fneur.2017.00744

**Published:** 2018-01-15

**Authors:** Junaid Bhatti, Barto Nascimento, Umbreen Akhtar, Shawn G. Rhind, Homer Tien, Avery Nathens, Luis Teodoro da Luz

**Affiliations:** ^1^Sunnybrook Research Institute, Sunnybrook Health Sciences Centre, University of Toronto, Toronto, ON, Canada; ^2^Sunnybrook Health Sciences Centre, University of Toronto, Toronto, ON, Canada; ^3^Defense Research and Development Canada (DRDC), Toronto Research Centre, Toronto, ON, Canada

**Keywords:** *N-*acetylcysteine, *N-*acetylcysteine amide, traumatic brain injury, neurofunctional outcome, animal models, oxidative stress, inflammation modulation

## Abstract

**Background:**

No new therapies for traumatic brain injury (TBI) have been officially translated into current practice. At the tissue and cellular level, both inflammatory and oxidative processes may be exacerbated post-injury and contribute to further brain damage. *N-*acetylcysteine (NAC) has the potential to downregulate both processes. This review focuses on the potential neuroprotective utility of NAC and *N*-acetylcysteine amide (NACA) post-TBI.

**Methods:**

Medline, Embase, Cochrane Library, and ClinicalTrials.gov were searched up to July 2017. Studies that examined clinical and laboratory effects of NAC and NACA post-TBI in human and animal studies were included. Risk of bias was assessed in human and animal studies according to the design of each study (randomized or not). The primary outcome assessed was the effect of NAC/NACA treatment on functional outcome, while secondary outcomes included the impact on biomarkers of inflammation and oxidation. Due to the clinical and methodological heterogeneity observed across studies, no meta-analyses were conducted.

**Results:**

Our analyses revealed only three human trials, including two randomized controlled trials (RCTs) and 20 animal studies conducted using standardized animal models of brain injury. The two RCTs reported improvement in the functional outcome post-NAC/NACA administration. Overall, the evidence from animal studies is more robust and demonstrated substantial improvement of cognition and psychomotor performance following NAC/NACA use. Animal studies also reported significantly more cortical sparing, reduced apoptosis, and lower levels of biomarkers of inflammation and oxidative stress. No safety concerns were reported in any of the studies included in this analysis.

**Conclusion:**

Evidence from the animal literature demonstrates a robust association for the prophylactic application of NAC and NACA post-TBI with improved neurofunctional outcomes and downregulation of inflammatory and oxidative stress markers at the tissue level. While a growing body of scientific literature suggests putative beneficial effects of NAC/NACA treatment for TBI, the lack of well-designed and controlled clinical investigations, evaluating therapeutic outcomes, prognostic biomarkers, and safety profiles, limits definitive interpretation and recommendations for its application in humans at this time.

## Introduction

Traumatic brain injury (TBI) is a leading cause of death and disability in the United States and globally (1, 2). In the USA, TBI results in more than 250,000 hospitalizations and 2.5 million hospital visits (3), and the costs of immediate TBI care is estimated to be up to 100 billion US$ (4). Moreover, the burden caused by the consequent degree of disability that TBI patients suffer is estimated to be $2.5–$6.5 million (5). These disabilities include, but are not limited to, severe motor and cognitive impairments and mental health problems, such as addiction and mood disorders (6).

At the brain tissue level, the damage from the primary insult is mostly irreparable (7, 8). Additionally, the initial tissue damage may be worsened by a complex secondary injury process following the primary insult (9). These processes consist of a cascade of metabolic, cellular, and molecular events related to extensive tissue destruction and repair (10). These mechanisms are represented by the imbalance of glucose demand and supply, disruption of calcium homeostasis, increased formation of free radicals, lipid peroxidation, mitochondrial dysfunction, and local release of catecholamines (11–13). It has been shown that these processes, or lack thereof, result in further damage to the already critically injured brain tissue (9, 14). Local consequences of this intricate process include vasoconstriction and formation of microthrombi in the microvasculature, with further ischemia and edema (15); initiation and exacerbation of peripheral and central inflammatory process with release of pro- and anti-inflammatory mediators (16); a subsequent rise of intracranial pressure (ICP) with unfavorable neurological outcome or death (17).

Part of this process, involving disruption of the capacity of mitochondria to scavenge free radicals or reactive oxygen species (ROS), is of particular interest in this review (18). The level of glutathione, a naturally available antioxidant within the mitochondria, decreases rapidly after brain tissue injury (19), which leads to accumulation of cytotoxic ROS. *N-*acetyl l-cysteine (NAC), a thiol containing l-amino acid, replenishes glutathione synthesis (20), and thereby may ameliorate secondary brain injury (20) as it counters the deleterious effects oxidative stress, promotes redox-regulated cell signaling, and dampens excessive immuno-inflammatory responses (21). NAC has been an FDA approved drug since 1985 (22) and has been used for management of acetaminophen toxicity (23). Additionally, a few clinical trials have evaluated NAC targeting neurological diseases, including autism (24), major depression and other psychiatry conditions (9, 25, 26), neonatal asphyxia (27), and neurodegenerative disease (28). Furthermore, recent studies have shown that NAC can reduce levels of oxidative-stress biomarkers following surgical trauma, such as in abdominal aortic aneurysm repair and surgical repair of atrial fibrillation (29, 30).

*N*-Acetylcysteine is relatively safe to administer, has mild side effects such as nausea, vomiting, rash, and fever, and rarely results in anaphylaxis (23). However, a limitation of using NAC is that it has a low blood–brain barrier (BBB) permeability (20). More recently, an amide derivate of NAC known as *N*-acetylcysteine amide (NACA) was developed with a higher BBB permeability than NAC resulting in increased central nervous system bioavailability (31). However, this new derivate has never been used in studies conducted in humans (32). Both NAC and NACA have not been approved for use in TBI by the FDA or Health Canada.

Given the lack of therapies shown to improve outcome following TBI, we sought to survey the current literature on the underlying the biological and clinical effects of NAC and/or NACA, with respect to their ability to improve neurofunctional outcome, *via* modulation of oxidative stress pathways, inflammatory responses, and cell death signaling in both humans and animals sustaining brain trauma.

## Methods

This systematic review was reported in accordance with the Preferred Reporting Items for Systematic Reviews and Meta-Analyses (PRISMA) guidelines (33).

### Search Methods

MEDLINE (1946–Nov 2017), EMBASE (1947–Nov 2017), Cochrane Controlled Trials Register, and Cochrane Database of Systematic Reviews (from inception to July 2017) were searched. The search was not restricted by date and language. Search terms were defined *a priori* and by reviewing the MeSH (Medical Subject Headings) terms of articles identified in preliminary literature searches. The search strategy was based on the initial Medline search strategy and was modified as necessary for the other databases. We used a sensitive search strategy combining MeSH headings and the keywords “acetyl-cysteine” or “acetylcysteine” or “cysteine hydrochloride” or “cystine l-cysteine” or “NAC” or “*N-*acetyl-B-cysteine” or “*N-*acetyl-l-cysteine” or “*N-*acetylcysteine” AND “brain injury” or “trauma.”

### Eligibility Criteria and Study Selection

We included experimental studies in humans or animals that measured the neurofunctional outcome (primary outcome) post NAC or NACA use in patients with TBI or in animal models of brain injury. To be included, studies should have performed standardized neurocognitive or behavioral tests to measure neurofunctional outcome. We also included studies that measured levels of biomarkers of oxidative stress, inflammation or cell death (secondary outcomes). Studies were required to have at least one comparator group without NAC administration or placebo, including before and after intervention comparison. We included human studies involving adult or pediatric patients. We excluded studies involving isolated spinal cord injuries, case reports, case series, and conference proceedings. Two of the review authors (JB and UA) not blinded to journal, institution or authors, independently screened the abstracts of identified studies, and determined the eligibility of each study. Each author screened the titles and abstracts of every record retrieved to determine which of the studies would be assessed further. If it was clear from the title and abstract that the article was irrelevant, the article was rejected. Full texts of the studies with questionable eligibility or considered eligible, were retrieved in this phase for evaluation. The reference lists of the retrieved articles were also searched for additional citations. In case of disagreement, consensus was reached by discussion with the senior author (LTDL).

### Interventions

In both human and animal studies, we included all regimens of NAC and NACA used (different loading and maintenance doses, intervals, duration and routes). Information about which placebo and its regimen was also retrieved. A summary of the characteristics of included studies is available in Tables 1 and 2.

**Table 1:** Characteristics of human studies.

Reference	Population, mean/median age	Sample size, % male	Injury type	Control	NAC(A) dose	*Via*	First dose	Other doses	Length of follow-up	Outcome(s) measured	Findings
Amen et al. (34)	Retired NFL players, age: NR	*N* = 30, 100%	Repeated mild TBI	Self-matched	Diet with NAC	Oral	NR	Diet supplement, dose NR	2–12 months	Microcognitive test, SPECT image analysis	Improvement of general cognition Improvements in brain perfusion

Hoffer et al. (35)	Military personnel, median age: 22 years	*N* = 81, 99%	Mild post blast injury	Placebo	4 g loading dose	Oral	Up to 72 h	4 g/day for 4 days followed by 3 g/day for 3 days	7 days	Hearing loss, headache, confusion, memory, sleep and balance problems, COWA, animal naming	Improvement in cognition and balance Significant resolution of symptoms

Clark et al. (36)	Children, mean age: 9 years	*N* = 14, 60%	Severe, GCS ≤ 8	Placebo	NAC 140 mg/kg/dose and probenecid 25 mg/kg/dose	NG tube	NR	NAC 70 mg/kg/dose, 17 doses over 3 days and probenecid 10 mg/kg 11 doses over 3 days	14 days	Adverse events and antioxidant reserve	No adverse events in the NAC group

*COWA, controlled oral word association test; GCS, Glasgow coma scale; NAC, N-acetyl cysteine; NR, not reported; SPECT, single-photon emission computed tomography*.

**Table 2:** Characteristics of 20 animal studies (21 experiments).

Reference	Animal model	*N*	Injury type	Control group(s)	Intervention	*Via*	Initiation of intervention	Other doses	Follow-up
Abdel-Baki et al. (37)	Sprague–Dawley rats, 250–300 g	NR	CCI, moderate	Sham injury + NS Injury and NS	NAC 150 mg/kg	IP	1 h	Once daily on days 1 and 2	1 week

Chen et al. (38)	Wistar rats, 250–300 g	51	Weight drop, moderate	Sham injury + NS Injury and saline	NAC 150 mg/kg	IP	15 min	Once daily on days 1, 2, and 3	3 days

Du et al. (39)	Long Evans pigmented rats, 360–400 g	74	Blast exposure, 14psi, mild	Normal control	NAC 300 mg/kg HPN-07 98.5%	IP	1 h	Twice daily on days 1 and 2	7, 14, and 21 days

Eakin et al. (40)	1. Sprague–Dawley rats. 350–400 g	26	FPI, 1.8–1.9 atm, mild	Sham Injury	NAC 50 mg/kg	IP	30 min	Once daily on days 1, 2 and 3	14 days

Eakin et al. (40)	ICR mice, 30–40 g	32	Weight drop, ~30 g, mild	Sham vehicle (DMSO) Sham + drug Injury	NAC 100 mg/kg	IP	1 h	NR	7 days
					Topiramate 30 mg/kg				30 days

Ellis et al. (41)	Cats	17	FPI, mild	Injury	1. Pre-TBI 326 mg/kg 2. Post-TBI 163 mg/kg	IP	30 min	NR	80 min

Ewert et al. (42)	Long Evans pigmented rats, 360–400 g	48	Blast exposure, ~ 14psi, mild	Injury	NAC 300 mg/kg HPN-07 300 g/kg	IP	1 h	Twice daily for 2 days	3 and 24 h 7 days and 21days

Gunther et al. (43)	Sprague–Dawley rats, 250–400 g	24	Penetrating ballistic like, moderate	Injury Sham surgery	NACA 300 mg/kg	IP	2 min	24 h survivors 300 mg/kg	2 and 24 h

Haber et al. (44)	Sprague–Dawley rats, 250–300 g	NR	CCI, moderate	Sham + NS Injury + NS Minocycline 45 mg/kg	1. NAC 150 mg/kg 2. NAC 150 mg/kg + minocycline 45 mg/kg	IP	1 h	Once daily on days 1 and 2	31 days

Hicdonmez et al. (45)	Sprague–Dawley rats, 280–320 g	36	Weight drop ~0.5J, moderate	(1) No injury (2) Injury	NAC 150 mg/kg	IP	15 min	NR	2 and 12 h

Kawoos et al. (46)	Sprague–Dawley rats, 300–350 g	88	Blast overpressure, mild	Placebo (6 groups based on repetitive BOP)	NACA 500 mg/kg in each group, for 6 different groups	IP	2 h in 6 groups and 15 min prior TBI in 1 group	1 group: NACA at 2 + 4 h post TBI	7 days

Naziroglu et al. (47)	Sprague–Dawley rats, 330 ± 20g	36	Weight drop contusion, moderate	No injury TBI TBI + Se	NAC 150 mg/kg	Oral	1 h	Once at 24 h, 48 h, and 72 h	3 days

Pandya et al. (48)	Sprague–Dawley rats, 300–350 g	51	CCI, moderate	TBI + vehicle	1. NAC 150 mg 2. NACA 150 mg/kg and 18.5 mg/kg/h	IP	5–30 min	18.5 mg/kg/h NAC, NACA or vehicle	25 h to 15 days

Senol et al. (20)	Sprague–Dawley rats, 300–340 g	36	Weight drop, moderate	No injury TBI TBI + Se	NAC 150 mg/kg	Oral	1 h	Once at 24 h, 48 h, and 72 h	4 weeks

Silva et al. (49)	Wistar rats,270–300 g	NR	FPI, moderate	Injury + NS	NAC 100 mg/kg	Oral	Immediately	Once daily for 5 weeks	5 weeks

Thomale et al. (50)	Sprague–Dawley rats, 300–350 g	48	CCI, moderate	Injury + NS	NAC 163 mg/kg	IP	Immediately	2 and 4 h	24 h

Thomale et al. (51)	Sprague–Dawley rats, 300–350 g	62	CCI, moderate	Injury + NS	NAC 163 mg/kg	IP	15 min	2 and 4 h	24 h

Xiong et al. (52)	Sprague–Dawley rats, 200–350 g	NR	CCI, moderate	Sham Injury Vehicle	NAC 163 mg/kg	IP	4 groups: 5 min before 30 min after 1 h after 2 h after	2 groups post TBI: 1. 5 m and 15 m 1. 5 m and 30 m	12 hours 14 days

Xiong et al. (53)	Sprague–Dawley rats, 200–350 g	NR	CCI, moderate	Sham Injury	NAC 163 mg/kg	IP	30 min	NR	1, 4 and 12 h 1 and 14 days

Yiand Hazell (54)	Sprague–Dawley rats, 200–350 g	64	FPI, moderate	Sham + NS Injury + NS	NAC 163 mg/kg	IP	5 min	6 and 24 h	6 and 24 h
Yi et al. (55)	Sprague–Dawley rats, 200–350 g	66	FPI, moderate	Sham + NS Injury + NS	NAC 163 mg/kg	IP	5 min	NR	6 and 24 h3 and 7 days

*atm, atmospheric pressure; BOP, blast overpressure; CCI, controlled cortical impact; DMSO, dimethyl sulfoxide; FPI, fluid percussion injury; HPN, 2,4-disulfonyl α-phenyl tertiary butyl nitrone; ICR, imprinting control region; IP, intra-peritoneal; NACA, n-acetylcysteine amide; NAC, n-acetylcysteine; NR, not reported; NS, normal saline; PSI, pounds per square inch; Se, selenium; TBI, traumatic brain injury*.

### Outcome Measures

The primary outcome in this review was the neurofunctional status of the participants after administration of NAC or NACA, compared with a control group, during the follow-up period established in each study. Several tests for assessment of different levels of neurocognition have been previously validated in the human and animal literature. For example, the use of novel object recognition in Morris Water Maze Task (57) for assessment of neurocognition, and Y-maze (40), for assessment of psychomotor skills, both used in animals. Other tests were used in humans, such as the MicroCog^®^—Assessment of Cognitive Functioning (MACF) (58), Controlled Oral Word Association test with animal naming (59), Romberg test (balance) (60), and the dynamic gait index (61). In addition, assessment of post-traumatic symptoms such as hearing loss, headache, dizziness, memory loss, and sleep disturbances were also conducted (35). The secondary outcomes were tissue biomarkers of inflammation, such as pro-inflammatory cytokines [e.g., interleukin (IL)-1β (62), tumor necrosis factor alpha (63)], neural injury [e.g., glial fibrillary acidic protein (GFAP) (64)], neurodegeneration [e.g., amyloid-β (64)], apoptosis (e.g., deoxy-nucleotide transferase dUTP nick and labeling) (38), and oxidative stress [e.g., cytosolic free Ca^++^, cytosolic ROS (65)].

### Risk of Bias Assessment

Risk of bias was assessed in duplicate for each study included. Any disagreement was resolved through discussion and consensus. Each included study was classified as a randomized controlled trial (RCT) or a non-randomized study. We assessed risk of bias in each human study incorporated describing the risks (low-risk, high-risk, and unclear risk) for selection bias, performance bias, detection bias, attrition bias, reporting bias, and other bias. For animal studies, we used the tool proposed by Krauth et al. (66) which includes randomization, allocation concealment, blinding, sample size, ethical compliance, statistical methods, outcome assessment, and follow-up.

### Analysis

Clinical and methodological heterogeneity across the studies were assessed by examining study design, details on subjects, baseline data, interventions and outcomes, to determine whether the studies were sufficiently similar or not. Large heterogeneity, and the absence of common outcome measures reported, precluded meta-analyses. Therefore, all studies were analyzed qualitatively with a descriptive systematic approach.

## Results

**Figure 1:**
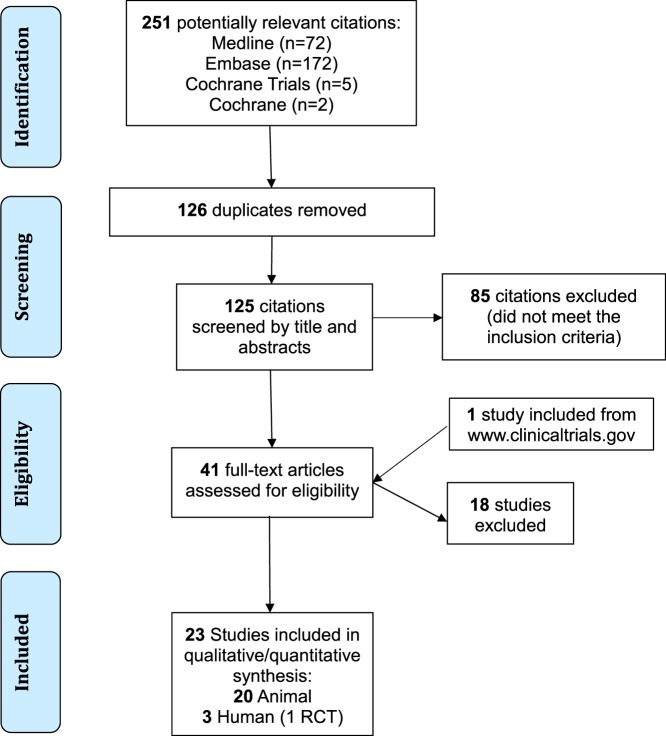
Flow diagram of the screening process. RCT, randomized controlled trial; TBI, traumatic brain injury.

The database search identified 251 potential studies for inclusion. After completion of the screening process, 23 studies were included in the qualitative analysis (20, 34–55). Three studies were conducted in humans (34–36) and 20 in animals (20, 37–45, 47–55). Other studies were excluded because they did not meet inclusion criteria, i.e., commentaries, conference abstracts, case reports and case series, or studies including spinal cord injuries. Figure 1 demonstrates the flow of the screening process. Studies excluded during the review process are reported in Supplementary SI in Supplementary Material.

### Clinical and Methodological Characteristics

The three studies conducted in humans were represented by two RCT (35, 36) and one observational cohort study (34) (Tables 1 and 2). The study with the largest sample size enrolled 81 active duty military personnel or veterans with blast-related mild TBI (35), whereas the other RCT recruited 14 pediatric patients with severe TBI (36). The observational study (34) was conducted in 30 retired professional football players who sustained repeated head impacts over extended periods of time with evidence of brain damage (mTBI/concussion) and cognitive impairment.

The 20 animal studies (20, 37–55) included 21 experiments with over 700 animals in total. Nineteen studies included rats (*n* = 700), however, five studies did not report sample size (37, 44, 49, 52, 53). One study examined mice (*n* = 32) (40), and one included cats (*n* = 17) (41). Sprague–Dawley rats were included in 15 studies (*n* = 491) (20, 37, 40, 43–48, 50–55). Studies used different brain injury models, such as controlled cortical impact in seven experiments (37, 44, 48, 50–53), weight drop in five experiments (20, 38, 40, 45, 47), fluid percussion injury in five experiments (40, 41, 49, 54, 55), blast exposure in three experiments (39, 42, 46), and ballistic-like TBI in one experiment (43). A moderate injury was inflicted on animals in 15 of these experiments (20, 37, 38, 43–45, 47–55) and mild injury in 6 experiments (39–42, 46).

### Interventions

Human studies used different regimens of NAC. For example, in the placebo-controlled RCT conducted involving military members (35), a loading dose of 4 g was administered orally within 72 h of mild TBI followed by 4 g/day for 4 days, and 3 g/day for 3 days. In the pediatric placebo-controlled trial (36), NAC was administered with probenecid with loading dosages of 140 and 25 mg/kg, respectively. A total of 17 maintenance doses of 70 mg/kg of NAC were administered over three days along with 11 maintenance doses of 10 mg/kg of probenecid. In the non-randomized trial (34), NAC was administered as one of the active agents of dietary supplements with no clarification of regimen.

In the animal studies, the loading doses of NAC ranged from 100–326 mg/kg with median dose of 163 mg/kg used in seven experiments (41, 50–55). In some experiments, other agents such as selenium (20, 47), 2,4-disulfonyl α-phenyl tertiary butyl nitrone (HPN-07) (39), topiramate (40), and minocycline (44) were used in combination with NAC. NACA, the BBB permeable derivative of NAC, was provided in three experiments (43, 46, 48). The route of administration in 17 studies (18 experiments) was intraperitoneal (37–46, 48, 50–55) and the drug was delivered *via* injection immediately after or up to 2 h post-injury (median 1 h). Subsequent doses were given in 16 studies, usually up to 48–72 h of the injury (20, 37–40, 42–44, 46–52, 54).

### Risk of Bias

Only the RCT conducted on military personnel following blast (35) had an appropriate design and sample size calculation required to detect differences between treatment groups (Tables 4 and 5). The pediatric RCT (36), though well controlled, had a small sample size of 14 patients and no sample size calculation was reported. Finally, the study conducted in retired professional athletes (34) was non-randomized, self-matched, and unblinded. This study reported limited information concerning attrition, the NAC regimen used, leading to a high risk of experimental bias.

**Table 3:** Outcome measures and summary of findings in 20 animal studies (21 experiments).

Reference	Outcome(s) measured	Summary of findings in *N-*acetylcysteine treated animals compared to controls
Abdel-Baki et al. (37)	Variants of place avoidance task (56)	1 NAC + minocycline: improvement of active place avoidance (F_4,25_ = 34.68; *p* = 0.01).
Chen et al. (38)	NH-kB; IL-1β; TNF-α; IL-6; ICAM-1 micro-vessels; brain water; BBB; cell apoptosis	1 NF-kB, UL-1β, IL-6, TNF-α, ICAM-1: upregulated following TBI and suppressed with NAC 2 NAC: ↓ Brain edema, barrier permeability, and apoptosis
Du et al. (39)	4-HNE; c-fos; amyloid beta (A4); APP; GFAP; NF-68; caspase 3	1 Blast exposure: upregulated 4-HNE, c-fos, GFAP, APP, and HNF 68 2 NAC: reduced the levels of all biomarkers in specific brain areas
Eakin et al. (40)	MWM: hidden platform, probe trial, visible platform	1 Treatment group: Improved performance in MWM tasks, probe trial, and visible platform tasks (*p* < 0.05)
Eakin et al. (40)	Novel object recognition; Y maze paradigm	1 Treatment group: significantly improved performance for both tasks (*p* < 0.05)
Ellis et al. (41)	Mean arterial pressure	1 Cats receiving NAC demonstrated vasoconstriction (one of mechanism involved in oxidative stress)
Ewert et al., (42)	ABR; DPOE level shifts; hair cell loss on cochlear histology	1 ABR threshold was 10 dB less at 24 h. Difference in thresholds was around 20 dB at 7 and 21 days between the treated and control group 2 DPOE levels shift recovered around 7d in treated group 3 Hair cell loss was significantly less in treated group
Gunther et al. (43)	Fluro-Jade B; TUNEL; MnSOD; Ox-42; iNOS; 3-NT; NFkB; Caspase 3; Cytochrome C; bcl-2; Cy3; Alexa 488; Biotinyl	1 Significant lower levels of necrotic cell death and apoptosis 2 Levels of antioxidant enzyme MnSOD were significantly higher
Haber et al. (44)	Four variants of place avoidance tasks (56). Brain sections of injured area	1 NAC + minocycline: performed better on conflict active place avoidance task and limited memory deficits 2 NAC + minocycline: associated with decreased CD68 expression and increased microglial activation.
Hicdonmez et al. (45)	MDA; SOD activity; GPx activity; catalase activityNumber of neurons (/mm^2^); caspase 3 activity	1 Treatment group: significant increased activity of SOD and GPx at 12 h 2 Treatment group: significant GPx activity at 2 h 3 Morphology of neurons was well protected in the NAC group
Kawoos et al. (46)	Intracranial pressure monitoring	1 Single NACA dose reduced ICP after single BOP induced injury 2 Single NACA dose was not effective in reducing ICP after multiple BOP 3 Two NACA doses were effective in reducing ICP after multiple BOP 4 A pre-injury NACA dose was most effective in reducing ICP after multiple BOP
Naziroglu et al. (47)	Intracellular Ca^2+^; apoptosis; caspase activity, and ROS in hippocampal cells	1 NAC group: ↓ Intracellular free Ca^2+^, apoptosis, caspase 3 and 9 activity, and ROS levels 2 Se group: some effect, but NAC group had a much greater effect
Pandya et al. (48)	Exp 1—tissue sparing; Exp 2—oxidative stress; Exp 3—mitochondrial bioenergetics and glutathione content; Exp 1–3 cognitive behavioral assessment	1 NACA: better than NAC for tissue sparing and cognitive behavior. 2 NACA: ↓ oxidative stress, improved mitochondrial bioenergetics, and maintenance of GSH
Senol et al. (20)	Lipid peroxidation; GSH; GSH peroxidase; protein assay; brain cortex β-Carotene, Vitamins A, C, and E	1 NAC group: significantly higher levels of GSH, Vitamin A, Vitamin E, and total oxidant status
Silva et al., (49)	Drug induced seizures; Na + K + ATPase activity; TBAR; protein carbonyl	1 NAC group: more protection from drug induced seizures and Na + , K + ATPase inhibition
Thomale et al. (50)	Brain edema, ABG; ICP; contusion volume	No differences were observed
Thomale et al. (51)	MAP; ABG; CBF; ICP; water content; contusion volume	No differences were observed
Xiong et al. (52)	Mitochondrial activity; Ca + 2 content and transport; GSH	1 NAC: significant impact on mitochondrial bioenergetics and Ca uptake 2 NAC: restored GSH levels during the 14-day observation period
Xiong et al. (53)	Apoptosis-related proteins; shortened bcl-2 and Bax; cytochrome c	1 NAC: diminished levels of shortened bcl-2 and Bax
Yi and Hazell (54)	HO-1 activity in specific brain regions; volume of injury	1 NAC: lower levels of HO-1 in cerebral cortex, thalamus, and hippocampus 2 NAC: associated with significantly decreased volume of injury
Yi et al. (55)	Complexin I and II; neuronal cell loss	1 NAC: reversed increased in Complexin levels in the injured cortex 2 NAC: significantly less neuronal cell loss

*HNE, 4-hydroxy-2-nonenal; SOD, superoxide dismutase; ABG, arterial blood gas; ABR, auditory brain stem; BB, blood–brain barrier; BOP, blast overpressure; CBF, cerebral blood flow; c-fos, genetic biomarker; COWA, controlled oral word association test; DPOE, distortion product optoacoustic emissions; GPx, glutathione peroxidase; HO-1, hemeoxygenase -1; ICP, intracranial pressure; ICAM, intercellular adhesion Molecule-1; IL-1β, interleukin 1-beta; IL-6, interleukin 6; MPO, myeloperoxidase; MDA, melandialdehyde; NAC, N-acetylcysteine; NACA, N-acetylcysteine amide; NH-kB, nuclear factor kappa B; ROS, reactive oxygen species; Se, selenium; SOD, superoxide dismutase; TBAR, thiobarbituric acid reactive substances; TBI, traumatic brain injury; TNF-α, tumor necrosis factor alpha*.

**Table 4:** Risk of bias in human trials assessing the role of *N*-acetylcysteine for traumatic brain injuries.

	Selection bias		Performance bias	Detection bias	Attrition bias	Reporting bias	Other bias
		
	Random sequence generation	Allocation concealment					
**Amen et al. (34)**							
Ranking	High-risk	High-risk	High-risk	Unclear	Unclear	High-risk	High-risk
Explanation	Not randomized	No allocation concealment	No blinding	No information provided	No information provided	Selective results presented	Other drugs used, no dosage, compliance not reported

**Hoffer et al. (35)**							
Ranking	Low-risk	Low-risk	Low-risk	Low-risk	Low-risk	Low-risk	High-risk
Explanation	Randomized	Allocation concealment done	Blinding of participants and assessors	Blinding of outcome assessor	All subjects followed to endpoint	Trial protocol and study reported	Generalizable? (conducted in the military setting)

**Clark et al. (36)**							
Ranking	Low-risk	Unclear	Low-risk	Low-risk	Low-risk	Low-risk	High-risk
Explanation	Randomized	No information provided	Blinding of participants and assessors	Blinding of outcome assessor	All subjects were followed to endpoint	Reported prior publication in clinicaltrials.gov	Small sample size

Ten animal studies had a random sequence of allocation (20, 38–40, 42, 43, 45, 47, 48, 51) and only 1 of them had allocation concealment and blinding (48). Other limitations of some studies included lack of reporting animal inclusion criteria, sample size calculation, and reporting of outcomes. For detailed information on each domain, please see Table 5.

**Table 5:** Risk of bias in animal studies.

Reference	Random allocation	Allocation concealment	Blinding	Inclusion/exclusion criteria	Sample size calculation	Compliance with animal requirements	Conflicts of interests disclosed	Statistical model explained	Animals without comorbidity	Test animal details	Every animal accounted	Dose-response model	Optimal time window used	Total
Abdel-Baki et al. (37)	No	No	No	No	No	Yes	Yes	Yes	Yes	Yes	No	Yes	Yes	7/13
Chen et al. (38)	Yes	No	No	No	No	Yes	No	Yes	Yes	Yes	Yes	Yes	Yes	8/13
Du et al. (39)	Yes	No	No	Yes	No	Yes	Yes	Yes	Yes	Yes	Yes	No	Yes	9/13
Eakin et al. (40)	Yes	No	No	No	No	Yes	Yes	Yes	Yes	Yes	Yes	Yes	Yes	9/13
Ellis et al. (41)	No	No	No	No	No	Yes	No	Yes	No	No	Yes	Yes	Yes	5/13
Ewert et al. (42)	Yes	No	No	No	No	Yes	Yes	Yes	Yes	Yes	Yes	Yes	Yes	9/13
Gunther et al. (43)	Yes	No	No	No	No	Yes	Yes	Yes	Yes	Yes	Yes	Yes	Yes	9/13
Haber et al. (44)	No	No	No	No	No	Yes	No	Yes	Yes	Yes	No	Yes	Yes	6/13
Hicdonmez et al. (45)	Yes	No	No	No	No	Yes	No	Yes	Yes	Yes	Yes	Yes	Yes	8/13
Kawoos et al. (46)	No	No	No	No	No	Yes	Yes	Yes	Yes	Yes	Yes	Yes	No	7/13
Naziroglu et al. (47)	Yes	No	No	No	No	Yes	Yes	Yes	Yes	Yes	Yes	Yes	Yes	9/13
Pandya et al. (48)	Yes	Yes	Yes	No	No	Yes	Yes	Yes	Yes	Yes	No	Yes	Yes	10/13
Senol et al. (20)	Yes	No	No	No	No	Yes	Yes	Yes	Yes	No	Yes	Yes	Yes	8/13
Silva et al. (49)	No	No	No	No	No	Yes	No	No	No	No	No	Yes	No	2/13
Thomale et al. (50)	No	No	No	No	No	Yes	Yes	Yes	Yes	Yes	Yes	Yes	Yes	8/13
Thomale et al. (51)	Yes	No	No	No	No	Yes	Yes	Yes	Yes	Yes	Yes	Yes	Yes	9/13
Xiong et al. (52)	No	No	No	No	No	Yes	No	Yes	Yes	Yes	No	Yes	Yes	6/13
Xiong et al. (53)	No	No	No	No	No	Yes	No	Yes	Yes	Yes	No	Yes	Yes	6/13
Yi and Hazell (54)	No	No	No	No	No	Yes	No	Yes	Yes	Yes	Yes	Yes	Yes	7/13
Yi et al. (55)	No	No	No	No	No	Yes	No	Yes	Yes	Yes	Yes	Yes	Yes	7/13
	10/20	1/20	1/20	1/20	0/20	20/20	11/20	20/20	18/20	15/20	14/20	19/20	18/20	

### Outcomes

#### Neurofunctional Status—Human Studies

The RCT conducted on military personnel (35) showed significant improvements in TBI symptoms, such as imbalance and headache, both assessed on day 7 in the treatment group compared to the placebo group (odds ratio 3.6, *p* = 0.006). The authors demonstrated that the proportion of symptom improvements was about 86% in those who were treated earlier with NAC (i.e., within 24 h post injury) as compared to 46% in those who received the drug between 24 and 72 hours. Significant improvements were from baseline values in NAC treated patients for trail making tasks A [*F*_(1,74)_ = 6.64, *p* < 0.05] and B [*F*_(1,74)_ = 4.87, *p* < 0.05]. Similar significant improvements were not seen in the placebo group. No significant differences were observed in hearing loss and memory problems between the NAC treated and the placebo group on day 7. The study on retired professional football players (34) showed significant improvements compared to their baseline measures in overall cognitive functioning (mean = 43 vs. 32, *p* < 0.001), cognitive proficiency (mean score = 35 vs. 25, *p* < 0.001), processing speed (mean score = 39 vs. 33, *p* < 0.001), processing accuracy (mean score = 49 vs. 41, *p* = 0.01), attention (mean score = 49 vs. 41, *p* = 0.01), reasoning (mean score = 42 vs. 33, *p* < 0.01), and memory (mean score = 43 vs. 34, *p* = 0.02). Improvements in cognitive functioning were associated with significant improvements in the brain perfusion (*p* < 0.001) in specific brain regions in the prefrontal, orbital, parietal, and occipital cortices. A recent phase 1 RCT conducted in 14 pediatric patients (36) reported no difference in the Glasgow outcome scale recorded upon hospital discharge or at three months follow-up.

#### Neurofunctional Status—Animal Studies

As compared to controls, animals treated with NAC showed significant improvements in specific neurocognitive and psychomotor tasks (Table 3). These tasks included active place avoidance (spatial memory) (37), novel object recognition (memory) (40), Y-maze (spatial memory) (40), probe trial (learning) (40), and visible platform tasks (visual acuity and motor ability) (40). In a study conducted in rats with moderate TBI (44) where NAC use was associated with minocycline, authors concluded that there was a possible synergy between the two drugs, leading to improvement of long-term memory and set-shifting, compared to controls. In another study where NACA was used (48), improvements in the acquisition phase of the Morris Water Maze task (spatial learning and memory) were demonstrated, compared to controls. Furthermore, NAC protected against hair cell loss that caused subsequent hearing impairment in another study (42). The effect of NAC on seizure disorder following TBI was addressed in a study with Wistar rats (49) and reported a reduced risk for pentylenetetrazol-induced seizures, compared to controls.

#### Biological Markers—Human Studies

In the most recent phase 1 RCT conducted in 14 pediatric patients (36), which evaluated the use of NAC and probenecid (pro-NAC), it was reported increased levels of both drugs in the cerebrospinal fluid of patients in the intervention group. The authors also measured levels of serum neuro-injury biomarkers, such as the Neuron Specific Enolase (NSE) and the Glial Fibrillary Acidic Protein (GFAP); however, levels of these biomarkers were not different between the intervention and control groups (NSE *F*[1,45] = 0.60, *p* = 0.441 and GFAP *F*[1,45] = 0.29, *p* = 0.596, respectively).

#### Biological Markers—Animal Studies

Recent animal experiments have focused on assessing the effects of NAC administration on levels of several oxidative stress biomarkers and glutathione (48) (Table 3). For example, several studies indicate better mitochondrial respiration and a higher glutathione content in animals with brain injury treated with NAC, compared to controls (48, 52, 53). Treatment with NAC was also associated with lower levels of IL-1β—a potent pyrogenic cytokine protein, nuclear factor (NF)-κB—prominent transcription factor that regulates inflammation and cellular survival, TNF-α—prototypical pro-inflammatory cytokine, IL-6—a key inflammatory and immunoregulatory cytokine, intercellular adhesion molecule-1—early adhesion protein that promotes leukocyte transmigration, 4-hydroxy-2-nonenal (4-HNE)—a marker of oxidative stress, c-fos—an immediate gene expressed in cell proliferation, regulation, and survival, GFAP—an astrocyte injury marker, the beta-amyloid precursor protein (β-APP)—a marker of chronic axonal damage, and neurofilament light—also a key marker of axonal injury (38, 39). Other studies have demonstrated improvements in makers known to modulate the oxidative stress, such as in apoptosis-related proteins [B-cell lymphoma-2 (bcl-2) protein and bcl-2-associated X protein (Bax)] (53), in a protein involved in the oxidative stress cascade [hemeoxygenase-1 (HO-1)], and in membrane proteins involved in neurotransmission (Complexin I and II) (55). Combined administration of NAC and Selenium was associated with reductions in cytosolic-free Ca^++^, apoptosis, cytosolic ROS, capsace-3, and capsace-9 (proteases responsible for the disassembly of the cell into apoptotic bodies), lipid peroxidation, total oxidant status, plasma IL-1β, and plasma IL-4 activities in rats inflicted with moderate TBI, as compared to untreated controls (20, 47).

*N*-Acetylcysteine amide has been used in three animal studies (43, 46, 48); two of which, examined biomarkers (43, 48) and one that reported its effect on ICP levels (46). Significant reductions in Fluoro-Jade (a marker of neuronal degeneration) and terminal deoxynucleotidyl transferase dUTP nick end labeling (TUNEL, a marker of apoptosis) were documented in Sprague–Dawley rats subjected to moderate TBI (43). The authors also demonstrated an increase in Manganese superoxide dismutase (MnSOD, an antioxidant enzyme) relative to controls (43). In another study (48), the use of NACA was associated with improvement of mitochondrial bioenergetics, glutathione content, cortical sparing, and reduced HNE levels.

### Other Secondary Outcomes Reported

Six studies (38, 41, 46, 50, 51, 54) assessed other secondary outcomes, such as the size of brain contusion, vasoconstriction or dilatation, ICP, edema, or imaging (Table 3). For example, in a study conducted in rats, use of NAC showed a non-significant decrease of 19% in contusion volume compared to untreated brains, as morphometrically measured using slice staining and imaging (50). The same investigators noted in another study with a larger sample size, that NAC treatment has no significant impact on ICP or water content (51). Similarly, the administration of NAC had no effects on cerebrovascular responsiveness as measured by intra-arterial pressure in brain vessels (41). At the cellular level, brain injury models where NAC was used for treatment, showed decreased brain edema, BBB permeability, and apoptotic cell death compared to untreated brains (38). Lastly, in a recent study, NACA significantly reduced the ICP in rats sustaining single and multiple injuries (two-way repeated measure ANOVA *p* < 0.05) (46). This study found that pre-injury and repeated doses of NACA were dose-dependently effective in reducing ICP after TBI (two-way repeated measure ANOVA *p* < 0.05).

### Adverse Events

No drug-attributable safety events were reported in any of the TBI studies cited. This conclusion is consistent with previous literature reports on the use of NAC for other medical conditions and as a potential performance enhancing ergogenic aid (67–69).

### Combined Therapies

The association of NAC with other drugs has demonstrated a potential synergistic effect in four animal studies included in this review (39, 40, 42, 44). Probenecid, well known to augment the systemic exposure of antimicrobial agents *via* inhibition of drug elimination through membrane transporters, can also prevent intracellular depletion of GSH (70). The study in children with severe TBI (36) has demonstrated that the co-administration of probenecid increases NAC concentration in both the brain and plasma, thus offering additive mechanisms for therapeutic synergy. In an animal model of blast injury (42) that produced auditory damage as primary sequela, a combination of an antioxidant (2,4-disulfonyl a-phenyl tertiary butyl nitrone [HPN-07]) and NAC could both enhance temporary auditory recovery and prevent permanent cellular damage when administered early post-blast exposure. The association of minocycline and NAC in a model of mild TBI (44) lead to a regulation of inflammation at tissue level (i.e., modulation of microglia), which may be an additional site of drug synergy between minocycline and NAC since microglial cell activation is known to be redox-regulated. Lastly, another experimental study where topiramate was administered in association with NAC (40) demonstrated the synergistic effectiveness of this combination as an adjunct for headache in a subgroup of animals. NAC alone resulted in a significant behavioral recovery after injury not affected by the use of topiramate.

## Discussion

### Main Findings

To our knowledge, this systematic review is the first to summarize and appraise the current evidence on the use of NAC in brain injury in human and in animal trials. The primary outcome of neurofunctional status, and the secondary outcomes of effect on markers of inflammation and oxidative stress at cellular and tissue levels and safety, were assessed to a limited extend in the included studies. Overall, NAC improved the neurofunctional status in humans and animals, reduced levels of mediators of oxidative stress and the inflammatory response at tissue and cellular levels, cell death, and had no safety concerns. The effect of NAC at tissue and cellular levels reducing inflammation and the oxidative stress has a potential to decrease secondary brain injury, which can affect positively the neurofunctional outcomes in brain injury patients.

Under physiological conditions, endogenous antioxidant systems maintain the redox homeostasis within the mitochondria to avoid accumulation of cytotoxic free radicals or ROS, which are formed as result of regular cellular respiration and metabolism. Brain injury causes dysregulation of this homeostatic process, with imbalance between ROS production and the cell’s antioxidant capacity, resulting in exacerbation of oxidative processes (71). The oxidative stress occurs within minutes of the primary mechanical impact (72) and is an important contributor to the pathophysiology of acute brain injury. Excitotoxicity provokes an excessive calcium uptake, reduces the membrane potential of mitochondria, increases production of ROS from the membrane enzyme complexes I and III, and subsequently reduces ATP production (18, 73). ROS initiate tissue damage by contributing to metabolic failure, to the breakdown of macromolecules, and to the oxidization of proteins, lipids and nucleic acids. Additionally, ROS enhance other secondary injury processes, including the excitotoxicity itself, inflammation, hyperadrenergic activation (13), mitochondrial dysfunction, which ultimately will lead to irreversible cell damage and death (72).

The recent interest in therapies targeting the damaged brain tissue, such as NAC, NACA, and the use of beta-blockers (74) are due to a better understanding of the pathophysiology of brain damage at tissue, cellular and molecular levels. Studies in animals and humans are focusing on identifying biomarkers of tissue damage, impaired metabolism, inflammation, oxidative stress, and cell death. Several biomarkers are currently associated with outcome in TBI and have been used as prognostic indicators. As an example, the hyperadrenergic response and catecholamine surge occurring in the early post-injury period following TBI was independently associated with unfavorable outcome and peripheral inflammatory cytokine/chemokine dysregulation in patients with moderate to severe isolated TBI (13, 16). Similarly, studies investigating the safety and efficacy of beta blockers in patients with acute TBI are currently ongoing (74).

Despite considerable investment by both the pharmaceutical industry and the US National Institutes of Health, to date, there are no pharmacotherapies that will definitively improve unfavorable outcomes post-brain injury. Human literature lacks properly designed phase 2 and 3 clinical studies evaluating new drugs, including NAC and NACA, as demonstrated in this review. There is a lack of studies that properly assess the effects of these promising compounds on the neurofunctional outcome, including cognition. Only one RCT (35) with an adequately powered sample size calculation was obtained. This study reported beneficial effects of NAC on patients with mild TBI-related balance disturbances and headache only. Conversely, a larger number of pre-clinical studies employing standardized brain injury animal models reported more convincing evidence of the utility of NAC/NACA. These models, previously validated in the animal literature (75) have demonstrated improvements in behavioral tasks related to memory, cognition, and auditory complications. Moreover, assessment of biomarkers of oxidative stress, inflammation (pro-/anti-inflammatory cytokines), and cell death (apoptosis-related proteins) was conducted mostly in the animal studies. They have demonstrated substantial modulation and downregulation of these pathways after the application of NAC. Evidence on other outcomes, such as the effect of NAC on brain perfusion, ICP, size of contusion, and brain reactivity remains modest to date.

### *N-*Acetylcysteine Amide

*N-*acetylcysteine amide is related to NAC with exception of a minor change in the chemical structure, an amide side chain substitution, that gives the compound a neutral charge and improves hydrophobicity and lipophilicity (76). As result of this minor modification, the efficacy of NACA as compared to NAC is significantly enhanced. The new physiochemical and pharmacological properties lead to an easier penetration into the BBB, mitochondria, and other cellular constituents. NACA seems to be a more attractive drug, with possibly stronger therapeutic properties for modulation of inflammation and oxidative stress post TBI due to its superior BBB permeability. It could be even more protective in TBI by simultaneously and effectively reducing the concomitant trauma-induced systemic inflammatory processes by reducing pulmonary injury (46). For example, in a study (77) where rats were administered with NACA and were exposed to a blast injury, there was a significant reduction of the infiltration of neutrophils into the lung and immunomodulation. NACA facilitated lung recovery from the inflammatory damage, which can be important in cases of severe ALI/ARDS and development of systemic inflammation that could further damage affect the already injured brain. Limited research on NACA has demonstrated that significant levels are detectable in the brain after oral and intraperitoneal administration, compared to NAC (21). If NACA is formulated as a co-crystal with an excipient, it may have a prolonged plasmatic half-life compared to NAC (78). Oxidative stress measures were reported in a model of intracellular oxidation in human red blood cells (79). In this experiment, NACA reduced oxidative activity five times more effectively than NAC and restored 91% of endogenous GSH compared to 15% with NAC, which may suggest an easier penetration of NACA through cellular membranes. The rationale for use of NACA in brain injury therefore seems robust. However, additional pre-clinical and clinical evidence is still required to better establish both mechanism of action and therapeutic efficacy.

### Strengths and Weaknesses of This Review

To our knowledge, no systematic review has been conducted in human and animal studies addressing the effects of NAC and NACA on neurofunctional outcome, tissue biomarkers of inflammation and oxidative stress, and safety in brain injury. Major limitations of this review are related to the weaknesses of many of the studies included. For example, it was not feasible to conduct a meta-analysis due to clinical and methodological heterogeneity across the studies, which subsequently precluded analysis of potential publications bias. Furthermore, in reviewing current evidence, the main limiting factor observed was that pre-clinical research on NAC and NACA in TBI is substantially more robust compared to clinical research. We identified several studies conducted in animals and only a few in humans. Pre-clinical work was mostly performed using previously validated animal models of brain injury; however, regimens of NAC and NACA administration, including dose (single vs. multiple), route (oral vs. intravenous), and time post injury still warrant better standardization. In addition, the effect of both drugs on other important biomarkers of brain and BBB damage predictors of outcome in TBI, such as the protein S100 beta (80, 81), need to be evaluated in pre-clinical and clinical studies. The use of NAC or NACA concomitantly with other drugs, such as the ones cited in this review, need more robust investigation to confirm whether synergistic or complimentary effects may occur. Moreover, the human literature presented in this review is restricted to small RCTs, not adequately powered to detect clinical differences, such as favorable or unfavorable neurofunctional outcome, mortality, and drug safety. Studies in humans also may lack generalizability, as they were conducted only in military personnel with specific blast injuries and in cases of pediatric neurotrauma.

### Unanswered Questions and Future Research

Although studies conducted in animal models of brain injury can provide insight and guidance to NAC and NACA use in TBI in patients, assessment and management of patients with TBI during clinical practice is considerably different from addressing TBI in these established animal models of brain injury (82). These differences may challenge the subsequent long-term outcome evaluation of NAC/NACA effects on TBI. Ultimately, findings from animal models must be translated and validated in the clinical setting, and this is importantly demonstrated in this review. Such knowledge translation strategies require major efforts and collaboration between clinicians and scientists, and can be challenging to achieve. However, those efforts are justified considering that TBI is a global leading cause of death and disability, have a substantial economic burden due the expenses in immediate and late care, and affects individuals regardless of age, sex, or race worldwide. Phase 3 and 4 trials are warranted for a better understanding of the efficacy and safety of NAC and NACA in TBI patients. Some questions still need to be answered in phase 1 and 2 trials. However, we believe that with the previous knowledge of efficacy and safety of NAC use in other clinical settings, a RCT is warranted. Futures studies should include a double-blind, placebo controlled, parallel trial, adequately powered to detect differences in laboratory and, more importantly, meaningful clinical endpoints, including neurofunctional outcome, mortality, and adverse events. We hope that this review will enable clinicians to better appreciate the current state of NAC/NACA use in TBI and prompt investigators to conduct well designed studies in future.

## Conclusion

In summary, our systematic review demonstrated moderate quality evidence of efficacy and safety of the use of NAC and NACA in pre-clinical studies. However, these studies still have important questions to be addressed and are substantially heterogeneous, which precludes a more robust interpretation. We found very limited clinical research addressing this subject in brain injury patients. Overall, the literature reported improvement of some aspects of neurofunctional outcome in human and animals, with decreased oxidative stress and inflammation at cellular and molecular levels, and no safety concerns. The promising effects of these drugs on the outcome of TBI warrant further animal research and translation to the clinical setting.

## Author Contributions

LL was the method expert responsible for study design, planning of data collection, data analysis, draft, and revision of the manuscript. JB and UA participated in the study design, data collection and analysis, and drafting of the manuscript. BN and SR were the content experts and participated in the study design, data analysis and revision of the manuscript. HT and AN participated in data analysis and revision of the manuscript. The final manuscript was approved by all authors.

## Conflict of Interest Statement

The authors declare that the research was conducted in the absence of any commercial or financial relationships that could be construed as a potential conflict of interest.
