# Supplementary material for: Systematic Review of Human and Animal Studies Examining the Efficacy and Safety of N-Acetylcysteine (NAC) and N-Acetylcysteine Amide (NACA) in Traumatic Brain Injury: Impact on Neurofunctional Outcome and Biomarkers of Oxidative Stress and Inflammation
Source: Front Neurol. 2018 Jan 15;8:744. doi: 10.3389/fneur.2017.00744 (PMC5776005; doi:10.3389/fneur.2017.00744)
Supplement: Supplementary file 1 [file data_sheet_1.PDF]

SEARCH STRATEGIES FOR NAC/NACA SYSTEMATIC REVIEW

Medline
Ovid Technologies, Inc. Email Service

Database: Ovid MEDLINE(R) Epub Ahead of Print, In-Process & Other Non-Indexed Citations, Ovid MEDLINE(R) Daily, Ovid MEDLINE and Versions(R) Search Strategy:
----
1 exp Acetylcysteine/ (12719)
2 (Acetyl Cysteine or Acetylcysteine or Cysteine Hydrochloride or Cystine L-Cysteine or NAC or N-Acetyl-B-Cysteine
or N-Acetyl-L-Cysteine N-Acetylcysteine).mp. [mp=title, abstract, original title, name of substance word, subject heading word, keyword heading word, protocol supplementary concept word, rare disease supplementary concept word, unique identifier, synonyms] (29667)
3 1 or 2 [NAC] (29667)
4 exp Brain Injuries/ (63787)
5 brain injur*.mp. [mp=title, abstract, original title, name of substance word, subject heading word, keyword
heading word, protocol supplementary concept word, rare disease supplementary concept word, unique identifier, synonyms]
(83847)
6 4 or 5 (90848)
7 trauma*.mp. [mp=title, abstract, original title, name of substance word, subject heading word, keyword heading
word, protocol supplementary concept word, rare disease supplementary concept word, unique identifier, synonyms]
(387677)
8 6 and 7 (47224)
9 6 and 7 [BRAIN INJURY] (47224)
10 exp Stress Disorders, Post-Traumatic/ (29004)
11 (post traumatic stress disorder or ptsd).mp. [mp=title, abstract, original title, name of substance word, subject
heading word, keyword heading word, protocol supplementary concept word, rare disease supplementary concept word, unique identifier, synonyms] (24063)
12 10 or 11 [PTSD] (36664)
13 3 and 9 (62)
14 3 and 12 (21)
15 13 or 14 (83)
16 remove duplicates from 15 (72)

****

EmBASE
Ovid Technologies, Inc. Email Service

Database: Embase Classic+Embase <1947 to 2017 Week 47> Search Strategy:
----
1 exp acetylcysteine/ (32397)

2 (Acetyl Cysteine or Acetylcysteine or Cysteine Hydrochloride or Cystine L-Cysteine or NAC or N-Acetyl-B-Cysteine
or N-Acetyl-L-Cysteine N-Acetylcysteine).mp. [mp=title, abstract, heading word, drug trade name, original title, device manufacturer, drug manufacturer, device trade name, keyword, floating subheading word] (47049)
3 1 or 2 [NAC] (47049)
4 exp brain injury/ (167383)
5 brain injur*.mp. [mp=title, abstract, heading word, drug trade name, original title, device manufacturer, drug
manufacturer, device trade name, keyword, floating subheading word] (144583)
6 4 or 5 (184418)
7 trauma*.mp. [mp=title, abstract, heading word, drug trade name, original title, device manufacturer, drug
manufacturer, device trade name, keyword, floating subheading word] (482121)
8 6 and 7 [BRAIN INJURY] (69512)
9 exp posttraumatic stress disorder/ (48746)
10 (post traumatic stress disorder or ptsd).mp. [mp=title, abstract, heading word, drug trade name, original title,
device manufacturer, drug manufacturer, device trade name, keyword, floating subheading word] (30031)
11 9 or 10 [PTSD] (52168)
12 3 and 8 (133)
13 3 and 11 (52)
14 12 or 13 (183)
15 remove duplicates from 14 (172)

****

CCRCT
Ovid Technologies, Inc. Email Service

Database: EBM Reviews - **Cochrane Central Register of Controlled Trials** <November 2017> Search Strategy:
----
1 exp Acetylcysteine/ (670)
2 (Acetyl Cysteine or Acetylcysteine or Cysteine Hydrochloride or Cystine L-Cysteine or NAC or N-Acetyl-B-Cysteine
or N-Acetyl-L-Cysteine N-Acetylcysteine).mp. [mp=title, original title, abstract, mesh headings, heading words, keyword]
(1727)
3 1 or 2 [NAC] (1727)
4 exp Brain Injuries/ (1167)
5 brain injur*.mp. [mp=title, original title, abstract, mesh headings, heading words, keyword] (3339)
6 4 or 5 (3425)
7 trauma*.mp. [mp=title, original title, abstract, mesh headings, heading words, keyword] (13362)
8 6 and 7 (2142)
9 6 and 7 [BRAIN INJURY] (2142)
10 exp Stress Disorders, Post-Traumatic/ (1261)
11 (post traumatic stress disorder or ptsd).mp. [mp=title, original title, abstract, mesh headings, heading words,

keyword] (2147)
12 10 or 11 [PTSD] (2456)
13 3 and 9 (4)
14 3 and 12 (1)
15 13 or 14 (5)
16 remove duplicates from 15 (5)

****

CDSR
Ovid Technologies, Inc. Email Service

Database: EBM Reviews - **Cochrane Database of Systematic Reviews** <2005 to November 16, 2017> Search Strategy:
----
1 (Acetyl Cysteine or Acetylcysteine or Cysteine Hydrochloride or Cystine L-Cysteine or NAC or N-Acetyl-B-Cysteine
or N-Acetyl-L-Cysteine N-Acetylcysteine).mp. [mp=title, abstract, full text, keywords, caption text] (79)
2 brain injur*.mp. [mp=title, abstract, full text, keywords, caption text] (308)
3 trauma*.mp. [mp=title, abstract, full text, keywords, caption text] (1901)
4 2 and 3 (199)
5 (post traumatic stress disorder or ptsd).mp. [mp=title, abstract, full text, keywords, caption text] (142)
6 1 and 4 (1)
7 1 and 5 (1)
8 6 or 7 (2)

****
